# Editorial: Sport performance analysis: from the laboratory to the field

**DOI:** 10.3389/fspor.2024.1372080

**Published:** 2024-02-02

**Authors:** Frédéric Meyer, Øyvind Sandbakk, Gregoire P. Millet

**Affiliations:** ^1^ Laboratory of Signal Processing 5, Swiss Federal School of Technology (EPFL), Lausanne, Switzerland; ^2^ Digital Signal Processing Group, Department of Informatics, University of Oslo, Oslo, Norway; ^3^ Department of Neuromedicine and Movement Science, Centre for Elite Sports Research, Norwegian University of Science and Technology, Trondheim, Norway; ^4^ Institute of Sport Sciences, University of Lausanne, Lausanne, Switzerland

**Keywords:** IMU (inertial measurement unit), wearable device, ecological condition, GNSS (global navigation satellite system), athlete monitoring

**Editorial on the Research Topic** Sport performance analysis: from the laboratory to the field

## Our research topic

Research in sport science has historically been performed in controlled laboratory conditions, as the analytical tools available were not portable or not adapted to field conditions. With the recent development in technologies and tools, a new field of research performed in more ecologically valid situations is emerging. This allows new insights to be generated and theoretical models of physiology and biomechanics studied in the laboratory to be validated for their application to the field.

The objective of this Research Topic was to provide a showcase for the development of new analytical methods adapted to the field. Furthermore, we wished to demonstrate how new technologies and analytical methods can be implemented in field conditions and provide relevant physiological and biomechanical information for analysis and performance enhancement in sport and exercise.

This editorial synthetizes findings from the four original articles included in this Research Topic. These were published in Frontiers in Physiology (Exercise Physiology) and Frontiers in Sports and Active Living (Elite Sports and Performance Enhancement), each contributing uniquely to the understanding of athletic development. Two papers delve into the comparative assessment of methods used in lab and field settings, while the other two provide novel insights into phenomena that elude observation in controlled environments.

## New insights

Van den Tillaar et al. aimed to compare sprint skating profiles among junior and senior bandy players of different playing positions. They used pairs of photocells placed every 10 m for an overall of 80 m to determine velocities and acceleration. Despite no differences in sprint skating performance observed between positions, senior players displayed notable distinctions compared to junior players. They were heavier, could accelerate faster, and reached higher velocities at an earlier time-point. The implications suggest a need for junior players to focus on power and sprint training to meet the demands of senior elite-level play. Together with other studies, where radar guns have also been used to determine instantaneous velocity and acceleration in hockey sprints (1), or to assess the validity of GNSS-IMU fusion algorithms (2), this provides new insights into field performance.

Du et al. compared 2,000 m and 3,000 m time trials (TTs) to estimate maximal aerobic speed (MAS) derived from laboratory-graded exercise testing (GXT) for collegiate runners. Utilizing ordinary least product regression, the study found that the 3,000 m time trial performance more closely approximated GTX-derived MAS. Time trials are simpler and less demanding to setup than GTX to prescribe training intensity. Nevertheless, bootstrap calibration of the equations was crucial, highlighting the necessity of detailed attention when using time trials as an alternative for MAS estimation.

Wei et al. investigated the effects of eight weeks of core stability training on freestyle skiing aerials athletes. They used a smart balance, specific core test, force platform and multi-cameras system to assess landing ability in the lab. The training group exhibited improvements in body shape, core stability, and landing kinetics compared to the control group. This study underscores the importance of targeted training, emphasizing the potential for enhanced sporting performance through specific interventions. In-field confirmation of the training effect could then be determined using dedicated force plates (3).

Finally, Seeberg et al. explored race development and performance-determining factors in mass-start cross-country skiing. The study combined global navigation satellite system (GNSS) with inertial movement sensors (IMUs) to monitor speed and related temporal patterns during a real-life competition with >100 competitors. They revealed novel data on mass-start events and showed the critical importance of starting position, incident avoidance, intensity tolerance, speed maintenance, and sprint abilities. These factors emerged as key determinants for success in mass-start competitions, highlighting the complexity of strategic decision-making during races.

## Concluding remarks

In the evolving landscape of sports science, these four papers showcase the importance of combining laboratory precision with real-world dynamics. Whether comparing assessment methods or uncovering unobservable phenomena, each study contributes to a richer understanding of athletic performance. These advancements not only inform training strategies but also pave the way for continued exploration at the intersection of scientific rigor and practical application in sports.
